# Evidence of Antibodies against the West Nile Virus and the Usutu Virus in Dogs and Horses from the Southeast of France

**DOI:** 10.1155/2023/8779723

**Published:** 2023-03-24

**Authors:** Younes Laidoudi, Guillaume Durand, Stéphanie Watier-Grillot, Anne-Sophie Dessimoulie, Claire Labarde, Thomas Normand, Virginie Andréo, Patrick Guérin, Gilda Grard, Bernard Davoust

**Affiliations:** ^1^Aix Marseille University, IRD, AP-HM, MEPHI, Marseille, France; ^2^IHU Méditerranée Infection, Marseille, France; ^3^National Reference Centre for Arboviruses, French Armed Forces Biomedical Research Institute (IRBA), Marseille, France; ^4^Unité des Virus Émergents (Aix-Marseille University-IRD 190-Inserm 1207-IHU Méditerranée Infection), Marseille, France; ^5^Animal Epidemiology Expert Group, French Military Health Service, Tours, France; ^6^Clinique Vétérinaire des 4 Chemins, Vic-La-Gardiole, France; ^7^1^st^ Veterinary Group, French Military Health Service, Toulon, France; ^8^41^st^ Veterinary Group, French Military Health Service, Fontainebleau, France; ^9^26^st^ Veterinary Group, French Military Health Service, Gramat, France; ^10^OpenHealth Company, Vannes, France

## Abstract

Every year, the world faces vector-borne diseases including arboviral (arthropod-borne viral) diseases caused by several, possibly fatal flaviviruses. The way they spread is related to a complex episystem involving several elements including vector abundance, animal carriers, and the flavivirus itself, which makes the disease difficult to manage. Here, we serologically screened 556 animals (358 dogs and 198 horses) using ELISA and a serum neutralisation test (SNT) for the anti-IgG antibodies directed against the West Nile (WNV) and Usutu (USUV) viruses. The animals investigated were split into two groups according to their exposure to the risk linked to the abundance of mosquitoes and migratory birds as well as the geographical distribution of arbovirus cases (458 animals from areas exposed to risk and 98 not exposed to risk). Overall, 25/310 dogs (8.1%) and 2/148 horses (1.3%) tested positive for SNT WNV and/or USUV in geographically exposed areas. Animals in unexposed areas were all negative. The geographical distribution of WNV seroprevalence in dogs was the same as the distribution of reported autochthonous human cases. Interestingly, a non-negligible seroprevalence caused by an as yet unidentified flavivirus other than WNV, USUV, or tick-borne encephalitis virus (TBEV) was detected in 18.6% (28/150) and 3.7% (4/106) of the investigated dogs and horses from the Hérault department, in the southeast of France, respectively. These data highlight the role of outdoor dogs as suitable sentinels for the evidence of WNV and USUV circulation in each area. In addition, the serological detection of an as yet unidentified flavivirus circulating in the Hérault department deserves greater attention, as this may constitute a serious threat to public and animal health.

## 1. Introduction

Vector-borne diseases, including arboviral (arthropod-borne viral) diseases caused by several possibly fatal flaviviruses, are responsible for the deaths of nearly 700,000 people in the world every year [1]. Since viral transmission takes place through mosquitoes, the epidemiology of arboviruses is linked to a complex episystem involving several factors including a favourable environment for vector proliferation, the reservoirs, and the arbovirus itself [2]. Fortunately, France has thus far been spared from the most serious arboviruses, despite increasing autochthonous outbreaks [3, 4].

The West Nile virus (WNV), a well-known flavivirus belonging to the Flavirideae family, was identified in 1937 in Africa [5, 6]. The first reported detection of the virus in France was in the southeast of the country, in the Camargue region, where an epizootic of equine encephalitis and some cases of human meningoencephalitis were noted between 1962 and 1965 [7]. Due to the continuous introduction of the virus through infected wild migratory birds, the natural carriers of WNV [8], and the presence of competent vectors (female *Culex* spp. mosquitoes) [9], several outbreaks and epidemiological foci of human and equine cases have been reported, including in the Alpes-Maritimes in 2003, the Pyrénées-Orientales in 2006, and 26 human and 13 equine cases in 2018 [10]. In 2022, in addition to dengue outbreaks, there were five equine cases of WNV, two bird cases of Usutu virus (USUV), and four human cases of WNV in the southeast of France [4, 11]. Like WNV, USUV is also transmitted by the bites of *Culex* spp. mosquitoes and spreads through wild migratory birds. USUV is not, however, pathogenic for horses. USUV was first identified in Africa in 1959 [12] and was recognised to be the causal agent of mortality in wild birds and of around 100 neurological human cases in Europe since 1996 [13]. The first French case was diagnosed in 2016 [14].

Regarding these two arboviruses, the French health authorities have implemented several measures for their epidemiological surveillance including virus detection in mosquito vectors, seroprevalence studies in birds including migratory species, and equine seroprevalence surveys and syndromic surveillance in horses [15–18]. Veterinary surveillance could be essential for estimating the risk for humans, but the surveillance of equines and birds is difficult to implement despite recommendations by authorities. Dogs are also considered excellent sentinels for the detection of viral circulation in each ecosystem [19–21]. To this end, the present study aimed to assess the recent seroprevalence (2021 and 2022) of both WNV and USUV in dogs and horses from three departments in the southeast of France (Hérault, Bouches-du-Rhône and Var), exposed to different epidemiological risks.

## 2. Materials and Methods

### 2.1. Sample Collection

**Figure 1:**
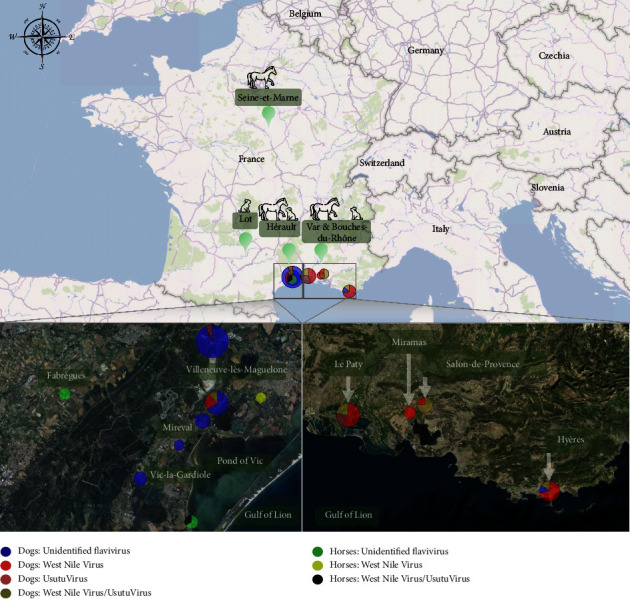
Map showing the geographical distribution of investigated dogs and horses. Pie chats represent the distribution of the 60 seropositive animals. Bubbles corresponding to municipalities are size-dependent according to the number of animals, and they are color-coded according to the infectious status of animals.

Between 2020 and 2022, 556 adult animals (358 dogs and 198 horses) were conveniently sampled from (i) exposed areas to WNV and USUV represented by three departments in southeast France, Hérault (150 dogs and 106 horses), Bouches-du-Rhône (138 dogs and 42 horses) and Var (22 dogs) and (ii) from nonendemic areas including 48 dogs from the Lot department (south-west) and 50 horses from Seine-et-Marne (north) which were used as control populations (Figure 1). These included 161 military working dogs (MWD), 117 shelter dogs, 34 breeding dogs, and 46 privately owned dogs (hunting dogs and pet dogs), as well as 50 military horses and 148 horses from equestrian leisure centres. No horses had been vaccinated against WNV.

All animals were subjected to blood sampling according to the best veterinary practices (no ethical issues arose related to animal suffering) as part of the epidemiological surveillance of canine and equine infectious diseases. Sera were collected in vacutainer dry tubes with a serum separator by centrifuging for 10 minutes at 3,500 rpm. Harvested sera were stored at −20°C until analysis.

### 2.2. Serological Detection

#### 2.2.1. ELISA

All animal sera were screened for the presence of IgG against WNV using an in-house indirect enzyme linked immunosorbent assay (ELISA) [22]. Briefly, the inactivated WNV supernatants from the French Centre National de Référence des Arbovirus (Marseille, France) were used as antigens for plate sensitisation. The anti-WNV IgG were revealed using a rabbit anti-dog and goat anti-horse IgG conjugate labelled with Fcy fragment-specific affinity-purified horseradish peroxidase (Jackson Immuno Research Europe Ltd.; Ely, Cambridgeshire, UK). Optical density was measured at 450 nm (Sunrise, Tecan Trading AG, Switzerland). The ELISA was interpreted relative to negative antigen (mock cell culture supernatant) as follows: ratio ≤3, negative; ratio >3, positive. All positive results were controlled by a neutralisation assay. For the serology of tick-borne encephalitis virus (TBEV), in-house IgG-capture enzyme immunoassay (direct ELISA) with whole inactivated TBEV was performed as previously described on 150 dogs from the Hérault department [23].

#### 2.2.2. Serum Neutralisation Test (SNT)

To confirm the ELISA results, neutralising antibodies were searched for using the microneutralisation assay. Briefly, 100 *µ*L of each ELISA-positive serum was 5-fold serially diluted from 1 : 20 to 1 : 360 in 96-well plates. Contacts were conducted at 50 median tissue culture infectious dose (TCID_50_) for one hour at 37°C in a 5% CO_2_ incubator. Both WNV (lineage 2, Austria 2016) and USUV (Senegal 1974) viral strains were used for the SNT assay. Finally, virus-antibody mixtures were added to Vero cells (American Tissue Culture Collection [ATCC] CCL-81, 1.3 × 10^5^ cells/well) and then incubated for four days. Cytopathogenic effects were investigated under a light microscope by a trained operator under biosafety level 3 (BSL-3) conditions. The neutralising titre was defined as the inverse of the highest dilution resulting in an infectious reduction of 50%. Given the cross-neutralisation between WNV and USUV, we interpreted the neutralisation assay as follows: (i) titre <1 : 20, negative; (ii) titre ≥1 : 20, for both viruses with a difference of more than two dilutions between them, has been interpreted as positive for the virus with the higher dilution and negative for the other; and (iii) all other situations have been interpreted as doubtful results.

### 2.3. Geographical Plotting of Positive Cases

All serologically positive samples were plotted according to the host and virus types using PowerBI software (https://powerbi.microsoft.com/fr-fr/).

## 3. Results and Discussion

Due to the high specificity rate of the serum neutralisation assay in discriminating between the possible WNV/USUV cross-reactions often occurring within ELISA [24–27], the SNT was considered the “gold standard” for definitive diagnosis of WNV infections [28]. Therefore, the present study followed the classical methodology based on SNT on sera from ELISA-positive animals.

None of the 48 dogs from Gramat (in the Lot department in southwest of France) or the 50 horses from Fontainebleau (in the Seine-et-Marne department in northern France) were ELISA-positive for any of the infections focused upon, highlighting the nonendemic nature of arboviruses in these regions, which is probably due to a lower vector pressure in these biotopes [29]. However, when it came to animals from the southeast of France, 21.3% (32/150) of dogs from Hérault, 16.5% (15/91) of those from the Bouches-du-Rhône living outside the city of Marseille, and 31.8% (7/22) of the dogs from the Var (Tables 1 and 2) scored positive using the ELISA assay. From the Hérault department, 5.6% (6/106) of horses were ELISA-positive. Seroprevalence clusters observed in the present study could be explained by the ecosystems and epidemiological pressure caused by the vectors. The Mediterranean-Rhodanian and Atlantic routes are the major axes of bird migration in France [30]. Gramat and Fontainebleau are outside these areas. In the areas of Gramat, Fontainebleau, and the city of Marseille, there are few freshwater ponds, and therefore, mosquito populations are greatly reduced as are migratory passages of birds, in contrast to the other sites in this study, which are highly exposed to these risks, especially due to the abundance of mosquitoes. For example, the Mediterranean coastal area represented in this study by the Hérault region features many small rivers, ponds, and abundant bird life (Figure 1). Similarly, Le Paty is in the Camargue wetland area (in the Rhone River delta), which is the focus for many migratory bird species and has experienced several outbreaks of West Nile virus since the 1960s [7, 31]. In the towns of Miramas and Salon-de-Provence, MWD are housed in kennels near small rivers. Finally, in the commune of Hyères, the kennels are located less than two kilometres from the salt marshes where many bird species have been observed, most of them migratory. Since the 1980s, these kennels are known to have been exposed to mosquito-borne diseases including dirofilariosis (i.e., 41% in Le Paty, 66% in Miramas, 88% in Salon-de-Provence, and 64% in Hyères) [32]. This may explain the epidemiological pressure caused by arboviruses, as they share the same Culicidae vectors. Recent studies showed that *Culex pipiens* is the most abundant species in these areas [9, 33].

Overall, 14, 4, and 7 dogs were SNT confirmed positive for WNV, USUV and WNV, and/or USUV, respectively. The SNT detected either WNV and/or USUV in four dogs (2.6%), 15 (10.8%), and six (27.2%) of the ELISA-positive samples from Hérault, Bouches-du-Rhône, and the Var, respectively. Only two (1.8%) of the six ELISA-positive horses from Hérault were detected by SNT. The seroprevalence by ELISA detected in dogs (21.3%) from Hérault was clearly higher than that reported in 2019–2020 from the same department (Montpellier, in Hérault), where a seroprevalence rate of only 1.6% (two USUV and one WNV/184 pet dog) was detected [18]. The dogs' lifestyle (pet dogs living indoors compared to kennel dogs in the present study) and the ecosystem of the study sites (an urbanised area compared to a humid coastal area in the present study) may explain the origin of this inconsistency in seroprevalence. It is obvious that tested working dogs living outdoors are more exposed to arboviruses than pet dogs housed indoors. In general, previous studies have highlighted a significant increase (from 1.4% to 14.6%) of flavivirus seroprevalence in birds between 2003 and 2019 in Hérault zoological park in Montpellier, with an increase in human cases over the 2016 to 2018 period [34]. In addition, compared to our previous flavivirus ELISA screening conducted in the Var department a decade before [19], there was a noticeable increase in seroprevalence in the same kennel (12% versus 31.8% in the present study), but this difference is not statistically significant (Chi2 test: *p* value = 0.09). Meanwhile, a consistent trend was observed for WNV seroprevalence over the last decade between the eastern coast of Corsica (8.4%) [20] and areas in the Bouches-du-Rhône (7.6%) given in the present study. Viral circulation therefore appears to be lower in the Bouches-du-Rhône than in the Var, a department where, in 2022, both equine and human cases were diagnosed [11]. These data highlight the clear increase of WNV and USUV seroprevalence over time, as reported in other areas of the Mediterranean Basin [13, 35, 36]. In Italy, especially in the north, in the Po River Valley, relatively close to southeast France, the annual incidence of human neuroinvasive WNV cases increased between 2012 and 2020, especially in 2018, with 448 cases reported during this period [37, 38].

Elsewhere in the world, in the United States, at the beginning of the West Nile epidemic affecting humans, horses, and birds, a seroprevalence (SNT) of 26% was recorded among 442 dogs [39]. In Serbia, which is one of the countries with the highest number of human WNV cases, in a survey carried out between 2011 and 2013, WNV seroprevalence according to SNT proved to be 36.9% in dogs (*N* = 184) and 34.9% in horses (*N* = 232) [40]. In Morocco, a study conducted on military dogs and horses showed seroprevalence of 62% (*N* = 231) and 60% (*N* = 349), respectively [41]. The present study revealed also the close relatedness between WNV seroprevalence among military dogs and horses sharing the same areas. From this information, it can be stated that the incidence of WNV and USUV in dogs can be predictive of infections in humans. Due to the threat caused by known or emerging arboviruses conducted by migratory birds from Africa [42], previous studies suggest the need to strengthen epidemiological supervision by including dogs having an outdoor lifestyle (i.e., MWD) as well as the classical targets such as horses and migratory birds [43].

Interestingly, a non-negligible seroprevalence of non-WNV and non-USUV infections was detected in 18.6% (28/150) and 3.7% (4/106) of the dogs and horses, respectively, investigated in Hérault. The ELISA test can detect flaviviruses in general, including WNV and USUV, but there may be other flaviviruses giving positive ELISA tests. This is why we carried out SNTs which confirm that there are seropositive animals for WNV, USUV, or other flaviviruses in the event of negative results to this test. In fact, these two techniques were used in the same way for the 310 dogs tested in the exposed area: 150 in Hérault and 160 in Bouches-du-Rhône and Var. We also tested all dogs from the Hérault department by IgG ELISA using TEBV as an antigen, and all were negative, whereas 32 were positive with ELISA using WNV antigen. We interpreted these ELISA results as flavivirus other than TBEV positive results and then tested the 32 positive sera using SNT against WNV (three positives) and USUV (one positive). In Hérault, 28/150 dogs were positive in the flavivirus ELISA, yet negative in the WNV and USUV SNTs. In the Bouches-du-Rhône and Var departments, only 1/160 dogs were positive in the flavivirus ELISA and negative in the WNV and USUV SNTs. The Chi2 test shows that there is a very significant difference between the two zones (*p* ≤ 0.0001). In Hérault, in dogs, the rate of exposure to an unknown flavivirus was 18.67%, whereas it was only 0.6% in the Bouches-du-Rhône and Var areas. These differences cannot be due to the methodology of analysis or the living conditions of the dogs because they were the same. They are most likely due to differences in the present ecosystems (mosquitoes and migratory birds), which are the determining factors in the transmission of the flaviviruses. ELISA cross-reactivity between WNV and other flaviviruses is not new, as was demonstrated previously for the tick-borne encephalitis virus [26]. TBEV is a common zoonosis in central Europe and Asia but remains nonendemic in the southeast regions of France. Furthermore, animals coming from the Hérault department in the present study were also subjected to a specific ELISA assay for TBEV, which detected only one seropositive horse and which did not cross-react with the WNV ELISA. Another flavivirus, namely, the Bagaza virus (BAGV), could be the origin for the ELISA seropositivity detected in the 28 animals from Hérault. BAGV has been known since 1966 in central Africa and was recently involved in fatal outbreaks among wild birds in Spain and Portugal [44]. The virus showed a strong cross-reactivity with both WNV and USUV on ELISA tests [25]. However, the circulation of other as yet unidentified flaviviruses in this area cannot be ruled out in the absence of deep molecular identification of flavivirus communities from birds and mosquitoes, and thus further molecular investigations and the isolation of other viruses are needed in this area.

## 4. Conclusion

Shelter and breeding dogs, particularly military dogs, are suitable sentinels for evidence of WNV and USUV circulation in a given ecosystem. Their periodic active serological surveillance should not be overlooked as a means to better determine the geographical spread of these arboviruses, thus predicting peaks of human and equine cases. In this study, we identified a common geographical distribution of seroprevalence in dogs and human cases. This highlighted the significant circulation of a non-WNV, non-USUV, and non-TBEV flavivirus in the Hérault department, which may constitute a serious threat to public and animal health. Genomic and viral isolation studies are urgently needed to identify the so far unidentified flavivirus involved.

## Figures and Tables

**Table 1:** Result of ELISA and serum neutralisation tests according to animal species and geographical origin.

Region	Department	Location	Date	Dogs						Horses					
				Number	ELISA flaviviruses positive (%)	SNT WNV positive (%)	SNT USUV positive (%)	SNT doubtful^*∗∗*^ (%)	SNT WNV and/or USUV positive (%)	Number	ELISA flaviviruses positive (%)	SNT WNV positive (%)	SNT USUV positive (%)	SNT doubtful^*∗∗*^ (%)	SNT WNV and/or USUV positive (%)
South East	Hérault	Mireval, Vic-La-Gardiole, Villeneuve-lès-Maguelone, Fabrègues	April 2022	150	32 (21.3%)	3 (2%)	1 (0.6%)	0	4 (2.6%)	106	6 (5.6%)	1 (0.9%)	0	1 (0.9%)	2 (1.8%)
	Bouches-du-Rhône	Marseille	June 2022	47	0					42	0				
		Le Paty (Camargues)	January 2021	45^*∗*^	9 (20%)	4 (8.8%)	3 (6.6%)	2 (4.4%)	9 (20%)						
		Miramas	January 2021	26^*∗*^	2 (7.6%)	2 (7.6%)	0	0	2 (7.7%)						
		Salon-de-Provence	January 2021	20^*∗*^	4 (20%)	1 (5%)	0	3 (15%)	4 (20%)						
		Total B-d-R		138	15 (10.8%)	7 (5%)	3 (2.2%)	5 (3.6%)	15 (10.8%)	42	0				
	Var	Hyères	January 2021	22^*∗*^	7 (31.8%)	4 (18.1%)	0	2 (9%)	6 (27.2%)						
	Total South East			310	54 (17.4%)	14 (4.5%)	4 (1.3%)	7 (2.2%)	25 (8%)	148	6 (4%)	1 (0.7%)	0	1 (0.7%)	2 (1.4%)

South West	Lot	Gramat (NE)	January 2021	48^*∗*^	0										

North	Seine-et-Marne	Fontainebleau (NE)	May 2022							50^*∗*^	0				

^*∗*^Military animals (i.e., dogs or horses) and ^*∗∗*^SNT doubtful where animals are positive for either WNV and/or USUV. NE: nonexposed sites to the risk of transmission.

**Table 2:** Concentration of anti-IgG antibodies by serum neutralisation testing among ELISA-positive animals. The value of the optical density of the ELISA. The infectious status and animal origins are provided.

Animal species	Department	Location	Code	ELISA flaviviruses POS D0 (%)	SNT WNV titer	SNT USUV titer	Interpretation
Horse	Hérault	Vic-la-Gardiole	EYG30	3.5	NEG	NEG	
			EYG44	3.3	NEG	NEG	
			EYG46	8.4	1/40	NEG	WNV
		Villeneuve-lès-Maguelone	EYG5	5.8	1/40	1/20	WNV and/or USUV positive^*∗*^
		Fabrègues	EYG60	3.9	NEG	NEG	
			EYG98	3.4	NEG	NEG	
Dog	Hérault	Mireval	CNH15	4.1	NEG	NEG	
			CNH16	3.4	NEG	NEG	
			CNH18	3.4	NEG	NEG	
			CNH19	3.6	NEG	NEG	
			CNH20	3.8	NEG	NEG	
			CVG2	3.4	NEG	NEG	
			CNH115	3.2	NEG	NEG	
			CNH116	4.6	NEG	NEG	
			CNH121	3.5	NEG	NEG	
			CNH129	11.2	1/80	NEG	WNV
			CNH132	5.7	1/80	1/20	WNV
			CNH135	12.2	1/40	NEG	WNV
			CNH142	3.1	NEG	NEG	
			CNH146	3.4	NEG	NEG	
		Vic-la-Gardiole	CNH5	3.4	NEG	NEG	
			CNH6	3.9	NEG	NEG	
			CNH7	3.4	NEG	NEG	
			CNH39	3.4	NEG	NEG	
			CNH45	3.8	NEG	NEG	
			CNH46	3.0	NEG	NEG	
			CNH50	4.2	NEG	NEG	
			CNH53	3.4	NEG	NEG	

		Villeneuve-lès-Maguelone	CNH56	3.1	NEG	NEG	
			CNH59	4.0	NEG	NEG	
			CNH61	4.1	NEG	NEG	
			CNH64	3.6	NEG	NEG	
			CNH71	3.6	NEG	NEG	
			CNH76	3.5	NEG	NEG	
			CNH77	3.2	NEG	NEG	
			CNH84	4.0	NEG	NEG	
			CNH85	3.3	NEG	NEG	
			CNH102	3.7	NEG	1/80	USUV
	Bouches-du-Rhône	Le Paty	SD10	7.7	1/40	NEG	WNV
			SD14	4.9	NEG	1/40	USUV
			SD15	12.6	1/40	NEG	WNV
			SD18	3.8	NEG	1/160	USUV
			SD24	4.6	NEG	1/40	USUV
			SD26	4.8	1/40	1/20	WNV and/or USUV positive
			SD31	12.1	1/80	1/20	WNV
			SD39	8.0	1/40	NEG	WNV
			I8	6.6	1/80	1/160	WNV and/or USUV positive
		Miramas	GA04	7.9	1/80	NEG	WNV
			GA26	6.8	1/40	NEG	WNV
		Salon de Provence	MM4	3.3	1/40	1/40	WNV and/or USUV positive
			MM5	3.5	1/20	NEG	WNV
			MM11	7.0	1/40	1/20	WNV and/or USUV positive
			MM12	5.2	1/40	1/40	WNV and/or USUV positive
	Var	Hyères	GF3	6.5	1/40	NEG	WNV
			GF9	7.5	1/160	1/80	WNV and/or USUV positive
			GF15	3.1	NEG	NEG	
			GF16	3.9	1/80	1/20	WNV
			GF18	3.3	1/40	1/20	WNV and/or USUV positive
			GF19	4.5	1/80	NEG	WNV
			GF20	4.9	1/80	1/20	WNV

^*∗*^Doubtful interpretation: animals were positive for either WNV and/or USUV.

## Data Availability

All data used to support the findings of the study are included within the article.
